# A Study of the Chemical Composition of the Dental Pulp

**Published:** 1889-12

**Authors:** W. H. Whitslar


### A Study of the Chemical Composition of the Dental Pulp.

BY W. H. WHITSLAR.

The dental pulp is a study, and we are constantly being impressed with the necessity of a thorough knowledge of all that pertains to the histological elements of it, but there seems to have been given little attention to the chemical composition of the pulp chamber by writers of the present day, and it is for a renewal of the subject that this study is made and presented for your consideration.

When we contemplate the small size of the dental pulp and remember that it is so closely bound by the hard and irresistible walls of dentine, is it any wonder that we find amongst our chemists very few who have ever attempted to analyze it? Indeed, it is a singular fact that there is no dental literature of recent origin, at least to my knowledge, that gives an analysis of the dental pulp. The only analyst that makes mention of such an examination was Prof. Wurtz, who in 1856 published in *L'Union Medicale* an article on the subject.

"Examined chemically by Professor Wurtz, it was found impregnated with a strongly alkaline liquid, and containing in solution a peculiar albuminoid substance. This substance must be a modification of the albumen which is formed by the action of the alkalies upon it. It is precipitated by acetic acid, and is thus distinguished from normal albumen. The liquid which holds it in solution is incompletely coagulated by heat; it is, moreover, precipitated by the mineral acids, tannin, and the metallic salts, such as the sub-acetate of lead, sulphate of copper, corrosive sublimate; alcohol coagulates it in thick flocculi. The alkalinity of this liquid excluding the idea of its holding the phosphate of lime in a state of simple solution; it appears more probable that this salt is intimately combined in the albuminoid matter itself."

The dental pulp reduced to ash gave, in the hands of the same chemist, a strongly alkaline residue, in which there was but a trace of phosphate of lime.

To Professor Wurtz, then, we give the credit of a very good analysis, but it must be borne in mind that the pulp is so small and heavily encased by the walls of dentine, as well as by the rather intimate union of the pulp by means of the odontoblastic attachment, so to speak, to the walls, that a percentage of the fluid contents of the pulp must be irretrievably lost in the extracting of the pulp from the chamber in which it lies. Again, an analysis of the ash of the pulp does not give the true composition of the salts as they existed in the living tissues.

The reasons are: 1st, when tissues are calcined a part of the salts may come from the oxidation of the sulphur and phosphorus of some of the organic constituents; 2nd, heat may decompose and volatilize certain chlorides and carbonates. Therefore, by making a chemical examination proper, much well worth knowing may be learned. Yet a *complete* analysis would be impossible. Willing to accept Prof. Wurtz's authority, we have made no chemical analysis of the subject.

Now, knowing the histological elements of the tooth pulp, and the chemical constitution of the separate elements, could we not make a nearer approach to the true composition chemically of the dental pulp than by a qualitative and quantitative analysis? Let us see.

The dental pulp is generally described as a roseate body—a mucoid gelatinous mass with abundant cells intermingled and including vessels and nerves. A fact right here is worthy of notice. Magitot states, and it is affirmed by Prof. Hutzman and Dr. Boedecker, that the dental pulp is the rudiment of the dental papilla and that it differs but slightly from the same organ in the foetus. Magitot says further that the calcareous masses and crystals of hæmatoidin noticed in the embryo are absent in the adult. Different authorities, including Magitot, Tomes, Wedl, and Black, say there are no lymphatics in the dental pulp, and this is controverted by Professor Stowell, who says: "The pulp is well supplied with lymphatics, their walls being formed by the endothelial membranes ensheathing the blood vessels." In an analysis of the pulp it is not important to include an analysis of lymph, because its composition is so near that of blood; and in fact, other tissues of the pulp include all the elements composing lymph, excepting sugar, which was found in human lymph by an analysis made by Gubler and Quevennel.

Along the border of the odonto-blastic layer of cells another study shows the deposition of lime salts, which, in their physico-chemical changes, are called calco-globulin, but when fully calcified produce phosphate and carbonate of lime, magnesia phosphate, and sodium chloride. These changes are also another means of making a real chemical analysis a failure, because as described by Tomes, "It is a remarkable fact that parts which are on the borders of calcification have a remarkable power of resisting reagents."

To sum up the tissues of the pulp there may be found the following:

Odontoblastic cells, connective tissue cells, nerve cells, medullary corpuscles, white fibrous and yellow elastic tissues, unstriped muscle fibres, cement substance for odontoblastic layer of cells, and cement substance for the connective tissues, lymph, blood and nerves.

Having learned the histological elements of the pulp, we may now inquire their chemical composition.

CELLS.—"Protoplasm is the essential constituent of the body of every cell." It is composed of water, albuminoid substances, and is associated with some carbohydrate, fat, and inorganic salts. Symbolically represented it is, $C_{24}$ $H_{17}$ $N_3$ $O_8$. The medullary corpuscles are composed principally of protoplasm.

White fibrous tissue is found as a constituent of every nerve and blood vessel and is distributed throughout the pulp. The chemical property of white fibrous tissue is Collagen, a body that furnishes gelatine when boiled in water. Mucin a nitrogenous glucoside is the material that composes the cement substance that holds together the connective tissue cells. Globulin is said to be the cement substance for the odontoblastic cells.

Nerves are said to enter the pulp canal by one large branch and three to six smaller ones. The fibres are medullated and non-medullated. "There is still much doubt and uncertainty as well as difference of opinion," says Cranston Charles, "as to the true chemical constitution of nerve substance, particularly as to which are real nerve constituents and which only decomposition products or mere mixtures." Without going into detail, it is sufficient to append the analysis of Maleschott, who gives the chief constituents of nerves, not brain or spinal cord, as follows:

| | |
|---|---|
| Water, | 57.07 |
| Ethereal extract, fat, cerebrin, cholesterin, lecithin, protogn, etc., | 22.11 |
| Salts, | 0.85 |
| Potassic phosphate, | 0.18 |
| Sodic phosphate, | 0.13 |
| Calcic phosphate, | 0.19 |

An analysis of the ash after the separation of lecithin, etc., by Geoghegan, gave

| | |
|---|---|
| Potassic chloride, | 0.277 |
| Sodic phosphate, | 0.221 |
| Potassic phosphate, | .047 |
| Sodic carbonate, | .044 |
| Magnesia phosphate, | .030 |
| Potassic sulphate, | .024 |
| Calcic phosphate, | .003 |

The blood and its vessels next command our attention. The *mean composition* of the blood as given by Becquerel and Rodier is

| | | |
|---|---|---|
| Water, | per cent. | 78.16 |
| Dry corpuscles, | | 13.50 |
| Albuminoids, | | 7.00 |
| Fibrin, | | 0.25 |
| Fats, | | 0.17 |
| Extractives, | | 0.84 |
| Earthy phosphates, | | 0.03 |
| Iron, | | 0.05 |

The ash of human blood is thus given by Jarisch in the 100 parts:

| | |
|---|---|
| Chlorine, | 30.74 |
| Potash, | 27.55 |
| Sodium, | 24.11 |
| Phosphoric acid, | 8.82 |
| Sulphuric acid, | 7.11 |
| Oxide iron, | 8.16 |
| Lime and Magnesia, | 1.33 |

In all analyses of blood or other circulating fluids it must be remembered that age, sex, and situation have much to do with their composition.

The blood vessels give us additional tissues to deal with, namely—unstriped muscular fibre and the yellow elastic tissue. The elastic tissue yields elastin, and the muscles yield water, albumen, ethereal extractives; non-introgenous bodies, glycogen and glycocin (Chittenden). The presence of unstriped muscle fibres and yellow elastic fibres have been denied by some.

In conclusion, we might add that all bodies of the dental pulp are alkaline; that the $CO_2$, N, O, in gaseous form would be found if it were possible to deal with a pulp on a gigantic scale; and that resolved into primary elements, the following would be found: Oxygen, Nitrogen, Hydrogen, Carbon, Phosphorus, Calcium, Sodium, Sulphur, Potassium, Chlorine, and Iron.

You will also notice that this is a study of the living dental pulp. A study of the dead and decomposing organ would be an altogether different thing.

Again, to be concise and not tiresome, many details of this paper are omitted for the sake of brevity alone, and having accomplished, in a measure, the study of the chemical composition of the dental pulp, it is hoped that this will prove an incentive to those who are better able to take up the subject to pursue it further.

